# Life factors acting on systemic lupus erythematosus

**DOI:** 10.3389/fimmu.2022.986239

**Published:** 2022-09-15

**Authors:** Jiaxuan Chen, Shuzhen Liao, Wanxian Pang, Fengbiao Guo, Lawei Yang, Hua-feng Liu, Qingjun Pan

**Affiliations:** Guangdong Provincial Key Laboratory of Autophagy and Major Chronic Non-communicable Diseases, Affiliated Hospital of Guangdong Medical University, Zhanjiang, China

**Keywords:** systemic lupus erythematosus, lifestyle, diet, smoking, alcohol, ultraviolet radiation

## Abstract

Systemic lupus erythematosus (SLE) is a highly heterogeneous autoimmune disease that primarily affects women. Currently, in the search for the mechanisms of SLE pathogenesis, the association of lifestyle factors such as diet, cigarette smoking, ultraviolet radiation exposure, alcohol and caffeine-rich beverage consumption with SLE susceptibility has been systematically investigated. The cellular and molecular mechanisms mediating lifestyle effects on SLE occurrence, including interactions between genetic risk loci and environment, epigenetic changes, immune dysfunction, hyper-inflammatory response, and cytotoxicity, have been proposed. In the present review of the reports published in reputable peer-reviewed journals and government websites, we consider the current knowledge about the relationships between lifestyle factors and SLE incidence and outline directions of future research in this area. Formulation of practical measures with regard to the lifestyle in the future will benefit SLE patients and may provide potential therapy strategies.

## Introduction

Systemic lupus erythematosus (SLE) is a highly heterogeneous autoimmune disease that primarily affects women, especially in the reproductive age. The prevalence rate of SLE worldwide is about 20–70 per 100,000 general population (1, 2). The exact etiology of SLE remains unclear, but genetic risk loci, such as N-acetyltransferase 2 (NAT2) slow acetylator genotype, and environmental factors are crucial in the development of susceptibility to SLE (3, 4). Although many SLE susceptibility genes have been identified recently, gene therapy approaches remain a distant prospect from the point of view of the clinical treatment (5). Furthermore, the significant side effects of high-dose immunosuppressive therapy for SLE, such as osteoporosis, hypertension and infection, have caused much concern (4, 6). Thus, the knowledge of environmental and lifestyle risk factors, especially those that can be controlled, may offer new promising therapeutic strategies for SLE.

Here we review evidence from reports published in reputable peer-reviewed journals and government websites and consider recent advances in our understanding of the links between lifestyle factors with SLE susceptibility and development. In particular, we analyze the effects of the 1) diet including N-3 polyunsaturated fatty acids (N-3 PUFA), N-6 PUFA, calorie restriction, vitamins, as well as 2) other lifestyle factors, including cigarette smoking, ultraviolet radiation exposure, consumption of alcohol and caffeine-rich beverages, etc. Implementation of practical measures with regard to these lifestyle factors will benefit SLE patients and may provide potential therapy strategies.

## Diet effects on SLE

### N-3 PUFA and N-6 PUFA

**Figure 1:**
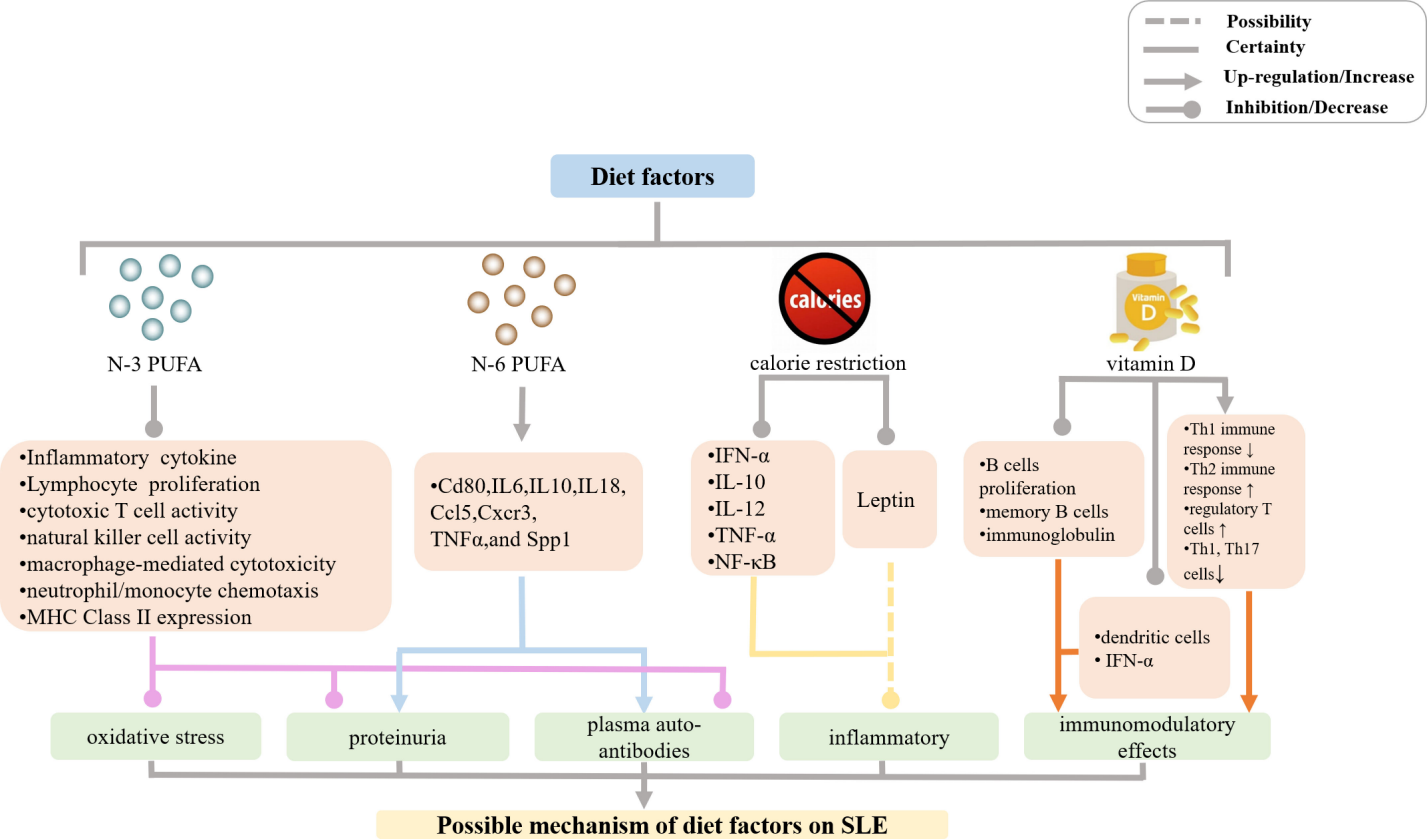
Mechanisms of diet effects on SLE incidence and manifestations. (PUFA, polyunsaturated fatty acids; MHC, major histocompatibility complex; IL, interleukin; IFN, interferon; TNF, tumor necrosis factor; NF, nuclear factor).

In the last thirty years, numerous studies in murine SLE models such as NZBWF1, BXSB/MpJ, and MRL-1pr/1pr mice reported that fish and olive oils containing N-3 PUFA effectively attenuated plasma auto-antibodies, proteinuria, and kidney glomerulonephritis as well as increased lifespan of animals, compared with the phenotypes of mice fed with beef tallow that contained saturated fatty acids, N-6 PUFA, or N-9 monounsaturated fatty acids (N-9 MUFA) (**Figure 1**) (7–12). Furthermore, an increasing number of human clinical trials demonstrated that consumption of N-3 PUFA had positive effects on autoimmune glomerulonephritis conditions, such as lupus nephritis and others (13–17). Since the earliest clinical trial in 1989, there have been seven major published clinical studies focusing on the relationship between N-3 PUFA and SLE. All but one of the clinical studies reported beneficial effects, including the improvement in endothelial function, disease activity, or inflammatory markers following the implementation of N-3 PUFA in SLE patients (18). A clinical nutritional study of SLE patients found that dietary patterns low in N-3 PUFA and high in carbohydrates positively correlated with the severity of disease activity, adverse serum lipids, and the presence of plaque (19). A double-blind, double placebo-controlled factorial trial in 52 patients with SLE (15) reported a significant decline in SLAM-R score (revised Systemic Lupus Activity Measure) from 6.12 to 4.69 in the subjects receiving eicosapentaenoic acid (EPA)/docosahexaenoic acid (DHA) compared to those on placebo. In the study carried out by Das and colleagues (20), daily oral supplementation of even moderate EPA and DHA (EPA 162 mg, DHA 144 mg) induced prolonged remission of SLE in ten patients. Furthermore, EPA and DHA also suppressed both T-cell proliferation and the production of inflammatory cytokines.

Mechanistically, N-3 PUFA prevented inflammatory and autoimmune responses mainly *via* anti-inflammatory and immune-modulating effects as it suppressed pro-inflammatory cytokine production, lymphocyte proliferation, cytotoxic T cell activity, natural killer cell activity, macrophage-mediated cytotoxicity, neutrophil/monocyte chemotaxis, MHC Class II expression, and antigen presentation (21–38). A large body of experimental evidence has shown that N-3 PUFA decreased plasma levels of interleukin (IL)-1β, IL-6, IL-10, IL-12, IL-18, tumor necrosis factor alpha (TNF-α), transforming growth factor beta 1 (TGF-β1), intercellular adhesion molecule 1 (ICAM-1), and fibronectin. N-3 PUFA increased the production of antioxidant enzymes and down-regulated mRNA expression of CD4^+^ T cell-associated genes, such as *Cd80*, *Il6*, *Il10*, *Il18*, *Ccl5*, *Cxcr3*, *Tnfa*, and *Spp1*, thereby reducing inflammatory response, oxidative stress, and autoimmune reactions in murine SLE models (11, 39–46). In contrast, N-6 PUFA-containing corn oil, safflower oil, and sunflower oil, which all induced the production of plasma auto-antibodies, proteinuria, and glomerulonephritis by increasing mRNA expression levels of the above-mentioned CD4^+^ T cell-associated genes in the kidney and/or spleen, contributed to the development of autoimmune reactions in NZBWF1 mice (11). The N-6 PUFA precursor was also shown to participate in the inflammatory process in SLE patients in a clinical study (13). However, the precise molecular mechanisms of N-3 PUFA and N-6 PUFA effects in SLE models remain unclear, and further studies are needed to confirm and correctly interpret the results of the published accounts.

### Calorie restriction

There have been many studies that examined the association between calorie restriction and autoimmune diseases such as SLE (**Figure 1**). Calorie restriction has been shown to alleviate SLE manifestations such as proteinuria, glomerulonephritis, and deposition of immune complexes as well as to prolong the lifespan of lupus mouse models by down-regulating mRNA expression of genes encoding the proinflammatory mediators IFN-α, IL-10, IL-12, TNF-α, NF-κB, and polymeric immune globulin receptor (47–52). This, in turn, reduced lymphoproliferation and antibody production, increased antioxidant defense, and decreased the extent of T lymphocyte shift (53–56). It is known that circulating levels of adipokine leptin markedly decrease with calorie restriction (57). Leptin has pro-inflammatory effects and may inhibit regulatory T cells as well as promote autoimmune responses (58–65). Hypoleptinemia and deficient leptin signaling led to the expansion of the population of regulatory T cells in NZB × NZW F1 mice (57), and a reduction in the number of Th17 cells in MRL/Mp-Faslpr mice (66), which contributed to the amelioration of SLE lesions. In addition, caloric restriction was also shown to significantly improve fatigue in subjects with SLE in a clinical study (67).

### Vitamin D

A large body of evidence in the last decade has suggested that vitamin D deficiency plays a key role in the development of autoimmune diseases such as SLE. Moreover, the degree of vitamin D deficiency in SLE patients correlates with the severity of SLE manifestations (**Figure 1**) (68–86). However, a study of a large prospective cohort of women born between 1980 and 2002 indicated that vitamin D consumption did not significantly affect the risk of SLE or rheumatoid arthritis (87). Furthermore, other prospective cohort studies suggested that dietary vitamin D intake during adolescence did not modify SLE risk in adulthood (88). Hiraki et al. suggested the association between dietary vitamin D intake and SLE risk may be misleading, because only 20% of vitamin D comes from food, whereas 80% of vitamin D is generated in the skin following exposure to UVB. Therefore, vitamin D consumption may not accurately reflect the extent of vitamin D deficiency or insufficiency (89). A clinical study conducted in 2017 showed that individuals with vitamin D deficiency are more prone to develop SLE compared with those relatives with SLE (90). In summary, there is a relationship between the degree of vitamin D deficiency or insufficiency and SLE incidence or exacerbation.

Immunomodulatory effects of vitamin D were examined in patients with SLE and it was then shown that 1,25-(OH)2-D3 suppressed the proliferation of activated B cells, decreased the number of memory B cells, and reduced the production of immunoglobulin, which also inhibited the maturation and activation of dendritic cells and reduced the production of IFN-α. In addition, 1,25-(OH)2-D3 also prevented Th1 immune response and simultaneously enhanced Th2 immune response, increased the number of regulatory T cells as well as decreased the numbers of Th1 and Th17 cells. These multiple effects lead to the recovery and maintenance of immune homeostasis, and an overall protective effect in SLE patients (86, 91–104). Although these observations justify the recommendation of vitamin D supplementation in SLE patients, the role of Vitamin D is not fully elucidated (105–107).

## Effects of cigarette smoking and consumption of alcohol and caffeine-rich beverages on susceptibility to SLE

### Cigarette smoking

**Figure 2:**
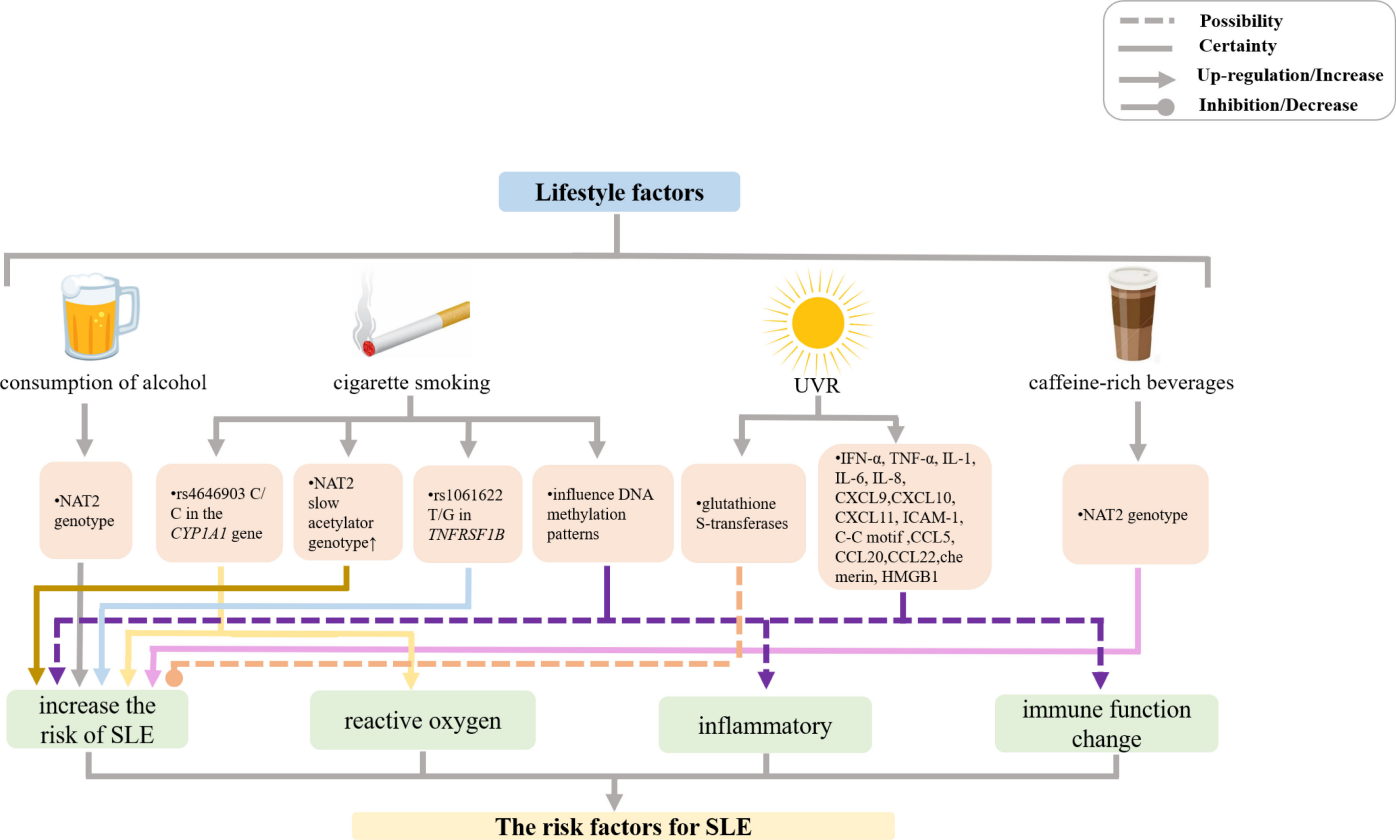
Mechanisms of other lifestyle factors effects on SLE incidence and manifestations. (UVR, ultraviolet radiation; NAT, N-acetyltransferase; IFN, interferon; TNF, tumor necrosis factor; IL, interleukin; CXCL, chemokine (C-X-C motif) ligand; CCL, chemokine (C-C motif) ligand; ICAM, intercellular adhesion molecule; HMG, high-mobility group protein).

Numerous epidemiologic studies revealed that exposure to cigarette smoke is associated with increased risk of SLE (**Figure 2**) (108–115). Furthermore, strong and consistent evidence suggests that current smoking is more risky than previous smoking (116–122). A study conducted by the Systemic Lupus International Collaborating Clinics/American College of Rheumatology Damage Index that involved 105 patients with SLE with 8.98-year follow-up indicated that smoking exposure may have deleterious effects on lupus morbidity (123). According to a meta-analysis conducted in 2004 that included seven case-control and two cohort studies, there was a modest association between current smoking and risk of SLE, whereas the effect of former smoking was not statistically significant (119). Subsequently, an updated meta-analysis in 2015, which contained 12 published articles encompassing 13 separate studies, found that the odds ratio (OR) values for SLE of current smokers and ex-smokers were 1.56 and 1.23, respectively, compared with the probability of SLE in nonsmokers (121). Recent research focused on cigarette smoking affecting clinical manifestations of patients with SLE has indicated that cigarette smoking was associated with photosensitivity, cutaneous damage, active SLE rash (124–127), higher SLE Disease Activity Index (SLEDAI) score (128), pleuritis, peritonitis, metabolic syndrome (129), neuropsychiatric symptoms (130, 131), vascular necrosis (132), thrombotic events (133–136), cardiovascular disease (137), peripheral vascular disease (138, 139), and production of anti-phospholipid antibodies (136). Moreover, smoking lowers the efficacy of medicines used to treat SLE (3, 140, 141). Likewise, a prospective cross-sectional study of Chinese SLE patients performed in 2015 reported that cigarette smoking causes the development and worsening of symptoms in SLE patients, including photosensitivity, nephropathy, proteinuria, compared with those in nonsmokers (after adjustment for age and gender), whereas SLEDAI scores were not significantly different in smokers and non-smokers (142). Taken together, these studies indicated that smoking is associated with increased risk for the development of SLE.

The mechanism whereby smoking affects SLE pathogenesis remains unclear. In recent years, several new lines of evidence have suggested that the effect of smoking in SLE may be modulated by gene polymorphisms and epigenetic changes. The studies of Japanese population by Kiyohara et al. showed that smokers with the N-acetyltransferase 2 (NAT2) slow acetylator genotype were at a significantly higher risk of SLE (OR 2.34, 95% CI 1.21–4.52) compared with nonsmokers carrying the rapid acetylator genotype (143). Moreover, Kiyohara et al. also demonstrated that smokers with rs1061622 T/G in *TNFRSF1B* that confers an increased risk for SLE (OR 1.56, 95% CI 0.99–2.47) had 49% of the excess risk for SLE resulting from the gene-environment interactions. In addition, although a significant association between the TT genotype of *STAT4* rs7574865 and increased risk of SLE (OR 2.21, 95% CI 1.10–4.68) was found in that study, there was no significant interaction between *STAT4* polymorphisms and smoking (144). Further, smokers carrying rs4646903 C/C in the *CYP1A1* gene that encodes a monooxygenase that generates various reactive oxygen species were also at a significantly increased risk of SLE (OR 9.72, 95% CI 2.73–34.6), as the presence of rs4646903 contributed over 60% excess risk of SLE (145). Therefore, several gene polymorphism-smoking interactions increase the risk of SLE. In addition, cigarette smoking, as a lifestyle factor, may influence DNA methylation patterns and thereby change the expression levels of disease-relevant genes (146–151). In a genome-wide DNA methylation analysis of peripheral blood mononuclear cells by Dogan et al., it was found that methylation levels of genes implicated in inflammatory and immune function pathways were altered by cigarette smoking, which could consequently cause complex illnesses with inflammatory components (152). Notably, there are indications that DNA methylation state may repair after the cessation of cigarette smoking (153, 154). However, much more remains to be done with respect to the elucidation of the interactions between gene polymorphisms and epigenetic changes on the one hand and smoking on the other hand.

### Ultraviolet radiation

Ultraviolet radiation (UVR) is an important environmental factor inducing SLE, as demonstrated in various studies of human populations and experimental studies (155) (**Figure 2**). It plays a crucial role in the pathogenesis of lupus by inducing a proinflammatory environment and leading to abnormal long-lasting photoreactivity *via* inflammatory mediators, such as proinflammatory cytokines, chemokines, and adhesion molecules. UVR exposure upregulates proinflammatory cytokines expression, such as IFN-α, IL-1, IL-6, and TNF-α (156). IFNs increase the expression of proinflammatory chemokines, including chemokine (C-XC motif) ligand (CXCL) 9, CXCL10, and CXCL11, which recruit chemokine (C-X-C motif) receptor 3 effector cells and induce keratinocyte apoptosis (157).

UVR also upregulates intracellular adhesion molecules, such as intercellular adhesion molecule 1 (ICAM-1) and lymphocyte function-associated antigen 1, and increases the secretion of chemokines, including IL-8, chemokine (C-C motif) ligand (CCL) 5, CCL20, CCL22, and chemerin, which are important for recruiting immune cells to areas of inflammation (158, 159).

In addition, one study revealed that UVR exposure induced high-mobility group protein B1 (HMGB1) release, which is related to the number of apoptotic cells in patients with SLE. HMGB1 released from apoptotic keratinocytes exerts inflammatory effects through binding to its receptors, resulting in the development of inflammatory lesions in the skin of patients with SLE upon UVR exposure (160).

If UVR is a trigger for SLE onset, glutathione S-transferases (GSTs, detoxification enzymes that protect cells from attack by reactive electrophiles that are produced by certain stressors, such as infection) may play a key role (3). The isoenzyme Mu of GST (GSTM1) is dominantly inherited. A population-based case-control study reported a threefold increased risk of SLE associated with 24 or more months of occupational sun exposure among Caucasian participants with the null GST Mu 1 (GSTM1) genotype (which leads to decreased activity of the GST enzyme). No effect of occupational sun exposure (on SLE risk) was seen in participants with the positive genotype (i.e., with the full activity of the GST enzyme) (161). However, more mechanisms of UVR affecting SLE disease progression need to be discovered and explored.

### Consumption of alcohol and caffeine-rich beverages

Previously, epidemiological studies showed that there was no significant association between alcohol consumption and SLE (110, 162–166). However, in the last several decades, several studies have consistently suggested that moderate alcohol consumption was negatively associated with the risk of SLE, irrespective of the type of alcoholic beverage (3, 112, 115, 167, 168). A meta-analysis of six case-control studies and one cohort study published in 2008 revealed that moderate alcohol consumption likely has a protective effect against the development of SLE (169). Furthermore, a case-control study from Japan suggested that consumption of black tea (OR = 1.88, 95% CI 1.03–3.41) and coffee (OR = 1.57, 95% CI 0.95–2.61) increased the risk of SLE (**Figure 2**) (170). Gene-environment interactions may be implicated in the mechanisms responsible for protective effects of alcohol consumption and SLE-aggravating action of caffeine-rich beverages. Kiyohara et al. showed that NAT2 genotype significantly affected the association between SLE risk on the one hand and alcohol and black tea consumption on the other hand (170). Another study that enrolled 505 patients with SLE from the Korean Lupus Network (KORNET) SLE registry between January 2014 and January 2016 showed that current alcohol consumption likely influenced the development of cutaneous damage in patients with SLE (166).

In conclusion, the available evidence reflects that cigarette smoking, caffeine-rich beverages, and UVR may promote the progression of SLE, while alcohol consumption is controversial and needs more research.

## Future directions

Modifying lifestyle risk factors could be the basis of potential preventative measures or therapy for SLE in the future. Insights into cellular and molecular mechanisms of negative and positive effects of lifestyle preferences on SLE incidence and manifestations are still being researched. These mechanisms involve gene-environment interactions, epigenetic changes, immune dysfunction, hyper-inflammatory response, cytotoxicity, and others. Practical measures with regard to these lifestyle choices in the future will benefit SLE patients and may provide potential therapy strategies.

## Author contributions

JC and SL wrote the manuscript and designed the figures. WP, FG, LY, H-FL, and QP revised the manuscript. All authors contributed to the article and approved the submitted version.

## Funding

This study was supported by the National Natural Science Foundation of China (no. 82070757, 81471530), the Department of established positions for the Zhujiang Scholar from Guangdong Medical University, and Guangdong Basic and Applied Basic Research Foundation (no. 2019A1515012203).

## Conflict of interest

The authors declare that the research was conducted in the absence of any commercial or financial relationships that could be construed as a potential conflict of interest.

## Publisher’s note

All claims expressed in this article are solely those of the authors and do not necessarily represent those of their affiliated organizations, or those of the publisher, the editors and the reviewers. Any product that may be evaluated in this article, or claim that may be made by its manufacturer, is not guaranteed or endorsed by the publisher.
